# Beyond the Usual Suspects: A Narrative Review of High-Yield Non-Traditional Risk Factors for Atherosclerosis

**DOI:** 10.3390/jcm15020584

**Published:** 2026-01-11

**Authors:** Dylan C. Yu, Yaser Ahmad, Maninder Randhawa, Anand S. Rai, Aritra Paul, Sara S. Elzalabany, Ryan Yu, Raj Wasan, Nayna Nanda, Navin C. Nanda, Jagadeesh K. Kalavakunta

**Affiliations:** 1 Department of Internal Medicine/Cardiology, Western Michigan University Homer Stryker School of Medicine, Beacon Kalamazoo Medical Center, Kalamazoo, MI 49007, USA; 2 Department of Internal Medicine, University of Alabama at Birmingham, Birmingham, AL 35233, USA; 3 College of William and Mary, Williamsburg, VA 23185, USA; 4 Plano West Senior School, Plano, TX 75093, USA; naynananda123@gmail.com; 5 Division of Cardiology, University of Alabama at Birmingham, Birmingham, AL 35233, USA

**Keywords:** atherosclerosis, atherosclerotic cardiovascular disease, non-traditional risk factors, South Asians, Asian Indians

## Abstract

**Background**: Cardiovascular risk models, such as the Framingham and atherosclerotic cardiovascular disease (ASCVD) calculators, have improved risk prediction but often fail to identify individuals who experience ASCVD events despite low or intermediate predicted risk. This suggests that underrecognized, non-traditional risk factors may contribute significantly to the development of atherosclerosis. **Objective**: This narrative review synthesizes and summarizes recent evidence on high-yield non-traditional risk factors for atherosclerosis, with a focus on clinically significant, emerging, and applicable contributors beyond conventional frameworks. This review is distinct in that it aggregates a wide array of non-traditional risk factors while also consolidating recent data on ASCVD in more vulnerable populations. Unlike the existing literature, this manuscript integrates in a single comprehensive review various domains of non-traditional atherosclerotic risk factors, including inflammatory, metabolic, behavioral, environmental, and physical pathways. An additional unique highlight in the same manuscript is the discussion of non-traditional risk factors for atherosclerosis in more vulnerable populations, specifically South Asians. We also focus on clinically actionable factors that can guide treatment decisions for clinicians. **Results**: Key non-traditional risk factors identified include inflammation and biomarker-based risk factors such as C-reactive protein or interleukin-6 levels, metabolic and microbial risk factors, behavioral factors such as E-cigarette use, and environmental or infectious risk factors such as air and noise pollution. We explore certain physical exam findings associated with atherosclerotic burden, such as Frank’s sign and Achilles tendon thickness. **Conclusions**: Atherosclerosis is a multifactorial process influenced by diverse and often overlooked factors. Integrating non-traditional risks into clinical assessment may improve early detection, guide prevention and personalize care. Future risk prediction models should incorporate molecular, behavioral, and environmental data to reflect the complex nature of cardiovascular disease.

## 1. Introduction

Cardiovascular disease (CVD) affects 621 million individuals worldwide, and two-thirds of CVD can be attributed to atherosclerosis [1]. Atherosclerosis, a chronic progressive, inflammatory disease characterized by arterial plaque buildup, underlies many cardiovascular conditions, including coronary artery disease (CAD), ischemic stroke, and peripheral vascular disease. Traditional risk factors for atherosclerosis include hypertension, dyslipidemia, diabetes, tobacco use, age, and family history. Despite decades of risk modeling, a substantial number of cardiovascular events occur in patients deemed low- or intermediate-risk by traditional tools such as the Framingham and Atherosclerotic Cardiovascular Disease (ASCVD) calculators [2]. This discrepancy suggests that non-traditional, under-recognized risk factors—ranging from immune dysregulation to psychosocial stress—play a greater role than previously understood.

Furthermore, certain populations are more vulnerable to cardiovascular disease, even in the absence of traditional risk factors. In this review, we seek to explore recent evidence on these populations and specifically highlight non-traditional risk factors for ASCVD in South Asians.

Despite their growing scientific support, many of these contributors remain underutilized in routine clinical risk assessment. This disconnect underscores the need for a comprehensive synthesis of high-yield, non-traditional atherosclerotic risk factors that are both clinically relevant and potentially modifiable.

In this narrative review, we aim to highlight the epidemiologically significant body of research for high-yield, non-traditional risk factors for atherosclerosis. We aim to provide clinicians, researchers, and healthcare policymakers with a practical, updated summary of these overlooked yet impactful contributors to ASCVD. This review article is distinct from prior ones in that it aggregates a wide array of non-traditional risk factors not listed in any single published piece of literature. In addition, previous review articles rarely synthesize recent research and evidence on ASCVD in more vulnerable populations, which we accomplish with this article. Lastly, this review paper seeks to translate emerging evidence into clear guidance for clinicians to risk-stratify patients and make treatment decisions, which distinguishes it from prior largely descriptive overviews.

## 2. Inflammation and Biomarker-Based Risk Factors

Atherosclerosis entails thickening of arteries with a decrease in lumen size, due to plaque formation. Cholesterol is the primary lipid component in atherosclerotic plaques. A crucial step in atherogenesis is the oxidation of low-density lipoprotein (LDL) and subsequent uptake by macrophages, which then become foam cells. These foam cells release pro-inflammatory cytokines that accelerate atherogenesis and plaque formation [3].

### 2.1. Lipoproteins, Apolipoproteins and Cholesterol Burden

There is a growing body of evidence in support of inflammatory and lipid-associated biomarkers involved in the pathogenesis of atherosclerosis. Historically, levels of total cholesterol have been heavily investigated and found to be associated with ASCVD. There is now a shift in focus to lipoproteins other than low-density lipoprotein (LDL). Among these, apolipoproteins have received significant attention, as they serve as an essential structural component to lipoproteins, in which cholesterol circulates.

Apolipoprotein B (ApoB), the structural protein of lipoproteins such as low-density lipoprotein (LDL) and very low-density lipoprotein (VLDL), has shown a robust and consistent association with cardiovascular events. ApoB particles traverse through arteries and can be trapped in arterial walls. While smaller ApoB particles contain less cholesterol, they bind more robustly with glycosaminoglycans in the arterial wall. In contrast, larger ApoB particles, which contain higher amounts of cholesterol, bind less robustly to the arterial wall, but once bound, end up releasing more cholesterol, thus resulting in damage [4]. As a result, ApoB particles, both small and large, are equally atherogenic. LDL can have high variability in cholesterol mass, which affects its atherogenicity. On the contrary, ApoB provides a more reliable estimator of atherosclerotic risk [5]. In a meta-analysis by Sniderman et al., ApoB was shown to be a more potent risk factor for cardiovascular risk than LDL cholesterol [6].

Lipoprotein(a) (Lp[a]) is a circulating lipoprotein that is atherogenic and a causal factor of CAD [7]. Lp(a) levels are affected by age, sex, lifestyle factors, and concomitant medical conditions. The *LPA* gene locus strictly controls Lp(a) levels [8]. The mechanism by which Lp(a) promotes atherosclerosis is not fully understood. Theories include the direct deposition of Lp(a) into the arterial wall. In addition, Lp(a) is thought to be more susceptible to oxidation than LDL, leading to the formation of foam cells. Lp(a) is also thought to induce endothelial dysfunction [9]. A 2025 retrospective study examined 484 patients with familial hypercholesterolemia (FH). They found that individuals with Lp(a) levels greater than or equal to 30 mg/dL were more likely to exhibit certain physical exam features, such as corneal arcus and increased Achilles tendon thickness. It is thought that apolipoprotein(a), a key component of Lp(a), has a high affinity for extracellular connective tissue components, resulting in these specific physical features. Recognition of this association has great clinical relevance in that FH patients with these physical exam findings should have an Lp(a) level checked [10], which can help predict ASCVD risk. Furthermore, elevated Lp(a) levels are associated with incident CAD even in individuals without first-degree relatives with heart disease [11].

Management of elevated Lp(a) remains a challenge and is actively being investigated. While a safe and effective treatment option is being investigated, the current literature suggests clinicians should address LDL levels and optimize all other modifiable risk factors [12].

### 2.2. ApoB/ApoA-I Ratio and Genetic Variants

The main structural protein component of high-density lipoprotein (HDL) is apoA-I. The balance between atherogenic and protective lipoproteins is often captured by the ApoB/apoA-I ratio. A meta-analysis by Forte et al. reported that individuals with peripheral artery disease (PAD) exhibited elevated ApoB levels and reduced apoA-I levels, yielding a significantly higher ApoB/apoA-I ratio—a superior predictor of atherosclerotic burden compared to LDL-C alone [13]. Zhang et al. retrospectively studied patients who had undergone percutaneous coronary intervention (PCI) and concluded that patients with an elevated ApoB/apoA-I ratio had a higher risk of major cardiovascular events one year post-PCI [14]. ApoB/apoA-I risk stratification goes beyond coronary atherosclerosis. For instance, in a study with type 2 diabetic patients, increased ApoB/apoA-I ratios were significantly associated with carotid atherosclerosis [15].

## 3. Inflammatory Cytokine Polymorphisms

Cytokines play an important role in the pathogenesis of atherosclerosis. For instance, in mice models with a genetic deficiency of tumor-necrosis factor alpha (TNF-a), there was demonstrated to be less uptake of LDL by macrophages and subsequent atherogenesis. TNF has been shown to be present in atherosclerotic plaques, and its levels are correlated with intimal thickness [16]. Therapy targeting TNF-alpha is the hallmark in managing conditions such as rheumatoid arthritis. In a study by Jacobsson, it was demonstrated that patients with rheumatoid arthritis treated with TNF blockers had a lower risk of developing CVD [17]. Immune cells, including macrophages, natural killer cells, neutrophils, and monocytes, also express interleukin-1 beta (IL-1β), a pro-inflammatory cytokine. In animal models, IL-1β accelerated intimal thickening [18]. Canakinumab is an IL-1β monoclonal antibody used in the treatment of select rare rheumatoid diseases. Ridker et al. demonstrated that canakinumab use led to a significantly lower rate of cardiovascular events compared to a placebo group [19]. While these studies demonstrate that anti-cytokine therapies can reduce cardiovascular events, their use in clinical practice for this purpose is limited.

Cytokine gene polymorphisms contribute to individual variability in inflammatory response and cardiovascular risk. For instance, a single-nucleotide polymorphism (SNP) in tumor necrosis factor-α’s(TNF-α)-1031C allele was found to be protective against atherogenesis in the carotid arteries [20].

### 3.1. Vasculitis

Systemic vasculitides are relatively autoimmune conditions that are associated with heightened inflammation. They result in immune-mediated injury ranging from small to large caliber blood vessels. Individuals with systemic vasculitides also tend to have concurrent systemic conditions such as hypertension and dyslipidemia, leading to increased ASCVD risk. Cardiac involvement can be seen in patients with vasculitides, particularly those such as polyarteritis nodosa and eosinophilic granulomatosis with polyangiitis. Accelerated atherosclerosis is thought to be secondary to vascular inflammation in conjunction with traditional cardiovascular risk factors. In addition, the mainstay treatment for many autoimmune conditions, glucocorticoids, is also linked with atherosclerosis. Sparse guidelines exist with regard to cardiovascular screening in patients with vasculitides [21]. Circulating biomarkers such as acute phase reactants and specific disease markers, such as antineutrophil cytoplasmic antibody, could be used to determine inflammatory burden and hence risk of atherosclerosis [22]. A 2023 study by Arevalo et al. compared ASCVD risk in patients with vasculitis compared to diabetes mellitus, a traditional risk factor. It found that patients with vasculitis were at higher risk of CVD and venous thromboembolism compared to patients with diabetes mellitus, emphasizing the importance of accounting for a patient’s vasculitis when stratifying their overall risk of ASCVD [23]. Appropriately controlling the underlying patient’s vasculitis is an essential component in their overall ASCVD risk-reduction.

### 3.2. C-Reactive Protein and Interleukin-6

Elevated C-reactive protein (CRP) and interleukin-6 (IL-6) levels can be seen in inflammatory conditions, and are implicated in the development and/or rupture of atherosclerotic plaque [24,25]. IL-6 is a regulator of inflammation and is found in atherosclerotic plaques. IL-6 has chemotactic properties that draw neutrophils and macrophages. This, in conjunction with the activation of endothelial cells, leads to atheroma production. In addition, IL-6 promotes the production and aggregation of platelets, leading to the formation of a thrombus. IL-6 also stimulates production of fibrinogen, which also amplifies platelet production. IL-6 can also promote the production of cellular adhesion molecules that result in the development of atherosclerosis [26]. On the other hand, CRP is an acute-phase protein also implicated in atherosclerosis. CRP is believed to hinder endothelial function and regeneration, leading to endothelial dysfunction and atherogenesis. CRP also activates and promotes diapedesis of leukocytes, leading to local inflammation [27].

Certain risk stratification tools, such as the Reynolds Risk Score, incorporate CRP levels in the assessment of cardiovascular risk [28]. Interestingly, certain therapies, such as statins, have been shown to reduce CRP levels [29]. Similarly, interleukin-6 (IL-6) is a proinflammatory cytokine that results in plaque formation and rupture [25]. A 2024 study analyzed IL-6 levels and cardiovascular mortality. They concluded that increased IL-6 levels were associated with increased all-cause mortality, in addition to worse cardiovascular outcomes [30].

### 3.3. Homocysteine

Homocysteine is a sulfur-containing amino acid thought to affect vascular inflammation through the promotion of oxidative stress [31]. In addition, homocysteine activates circulating leukocytes and platelets as well as stimulates vascular smooth muscle cell proliferation, leading to atherogenesis. Homocysteine can lead to expression of chemokines and adhesion molecules, resulting in circulating inflammatory cells adhering to the arterial wall [32]. In a 2006 study, homocysteine levels were associated with coronary artery calcium (CAC) scoring [33]. More recently, a 2020 study examined patients in the Multi-ethnic Study of Atherosclerosis (MESA) cohort. They concluded an association between homocysteine levels and incidence/progression of coronary calcifications [34]. The use of combined folic acid and B12-vitamin supplementation can be useful in decreasing homocysteine levels [35]. These vitamins serve as cofactors for the enzyme methionine synthase, which converts homocysteine into methionine and removes it from circulation.

### 3.4. Immune Dysfunction

A maladaptive immune response can lead to the initiation and progression of atherosclerosis [36]. Circulating monocytes are associated with severity and plaque size in atherosclerosis. The inhibition of monocytes in mice models leads to decreased atherogenesis [37]. Macrophages also promote atherogenesis in humans and actually play a role in every artery plaque formation [38]. At the onset of arterial damage, neutrophils are recruited to the site and induce a strong inflammatory response, thereby leading to atherosclerosis [39].

## 4. Metabolic and Microbial Risk Factors

### 4.1. Hyperuricemia

The end product of purine metabolism is uric acid. Hyperuricemia can be triggered by the intake of rich purine foods, alcohol intake, and consumption of high-fructose-containing foods. Hyperuricemia has been increasingly recognized as an independent contributor to atherosclerosis, primarily through its pro-inflammatory and oxidative effects on the endothelium, resulting in plaque formation [40]. For instance, intracellular uric acid is thought to promote oxidative stress via the formation of reactive oxygen species. In addition, uric acid itself can promote the production of inflammatory cytokines, chemokines, and adhesion factors, leading to the formation of atherosclerotic plaque [41]. In a large cohort study in Asia, Cui et al. demonstrated that uric acid is a stronger prognostic indicator in patients with no or few standard modifiable cardiovascular risk factors, such as diabetes, tobacco use, hypertension, and hyperlipidemia, compared to individuals with greater than two standard modifiable risk factors [42]. While current guidelines recommend treating symptomatic patients with hyperuricemia, treatment in asymptomatic patients is less clear and requires further investigation. Symptomatic patients, such as those with gout, may be treated with medications such as xanthine oxidase inhibitors, and uricosuric agents, uricase, as well as with risk factor modification, etc. [43].

### 4.2. Gut Microbiota and Cardiometabolic Effects

The gut microbiome plays a critical role in regulating host metabolism, inflammation, and immune responses. Microbial-derived metabolites—including short-chain fatty acids (SCFA), bile acids, and trimethylamine N-oxide (TMAO)—modulate lipid and glucose metabolism and can influence vascular tone and endothelial function. TMAO is traditionally thought to be pro-atherogenic [44]. During metabolism of phenylalanine, the gut microbiome produces phenylacetylglutamine (PAG), which can promote adverse cardiovascular phenotypes [45]. It is believed that exercise interventions can modulate the gut microbiome and reduce gut inflammation, thereby minimizing atherosclerotic risk [46].

### 4.3. Vitamin D Deficiency

Although the role of vitamin D in vascular health has long been debated, accumulating evidence suggests a relationship between deficiency and subclinical atherosclerosis. Vitamin D is thought to inhibit endothelial cell dysfunction and downregulate inflammatory processes, thereby slowing atherosclerosis [47]. Clinical trial data on the effects of vitamin D on atherosclerosis are more ambiguous, and as such, there are no clear clinical guidelines on this matter [48]. Recent data, including findings published in Desai et al., indicate that supplementation alone may not improve endothelial function, pointing instead to a complex, possibly nonlinear role of vitamin D in atherogenesis and also the need for further research on this topic [49].

## 5. Behavioral and Physiologic Risk Factors

### 5.1. Obstructive Sleep Apnea (OSA)

OSA is increasingly implicated in the pathogenesis of atherosclerosis due to its associations with oxidative stress and systemic inflammation. In animal models, intermittent hypoxia has been shown to trigger atherogenesis [50]. Particularly, intermittent hypoxia can lead to blood pressure augmentation, particularly diastolic pressures during hypoxic events. This is thought to lead to hemodynamic strain on the vascular wall, resulting in shear stress and subsequent inflammatory conditions and vascular remodeling, leading to atherosclerosis. In addition, OSA hypoxia can lead to the activation of the transcription factor, nuclear Factor kappa B, resulting in pro-inflammatory effects [50]. Notably, atherosclerosis has been found in patients with OSA without other traditional cardiovascular risk factors, and the severity of nocturnal hypoxia is directly correlated with atherosclerotic burden [51,52]. Dziewas et al. examined 214 patients with ischemic strokes and found that individuals with OSA were more likely to have atherosclerotic lesions in their carotid arteries [53]. Weinrech et al. noted a relationship between increasing apnea–hypopnea index (AHI) and CAC scoring [54]. Inflammatory mediators such as CRP and serum amyloid A are essential in the inflammatory cascade of OSA. Treatment of OSA with continuous positive airway pressure (CPAP) has not been consistently shown to decrease inflammatory mediators [55]. However, treatment with CPAP has been shown to decrease the progression of atherosclerosis [56].

### 5.2. Depression, Anxiety and Psychosocial Stress

Depression is associated with risk of cardiovascular disease. In depression, certain biological processes occur that lead to atherosclerosis, including decreased myocardial perfusion and noradrenergic hyperactivity [57]. While the specific pathogenesis of atherosclerotic plaque in depression is still being investigated, a 2025 review and meta-analysis clearly demonstrated that individuals with severe depressive symptoms had increased levels of CRP, IL-6, and TNF-a [58]. Saleh et al. also demonstrated that depression was associated with increased carotid intima-media thickness (CIMT) [59]. Scierka et al. reported a 24% increased risk of all-cause mortality among patients with PAD and comorbid depression [60]. Similarly, Brostow et al. noted that depression in PAD patients was associated with worsened ambulation and impaired lower extremity function, suggesting a bidirectional relationship between vascular insufficiency and mental health [61].

Post-procedural outcomes also appear affected. Zhang et al. reported a 57% increased risk of death or major adverse cardiovascular events (MACE) in patients with depression following percutaneous coronary intervention (PCI) [62]. Wu et al. [17] further demonstrated that depressive symptoms correlate with markers of subclinical atherosclerosis, including elevated CIMT, increased pulse wave velocity (PWV), and reduced flow-mediated dilation (FMD) [63]. Khan et al. reinforced these associations in CAD-free individuals, identifying a link between depression and CAC, fulfilling Bradford Hill causality criteria such as temporality and dose–response [64].

Although less extensively studied, anxiety disorders may also influence cardiovascular outcomes. Tully et al. found a significant association between generalized anxiety disorder (GAD) and MACE among outpatients with CAD [65]. Collectively, these findings emphasize the need to integrate mental health screening and intervention into cardiovascular risk assessment and management.

### 5.3. Alcohol Consumption

Alcohol exerts a dose-dependent effect on cardiovascular health. While traditionally light-to-moderate intake had been proposed to confer cardioprotection in some studies, higher levels of consumption are clearly deleterious. Notably, more recent research suggests that alcohol use at all levels is actually associated with increased cardiovascular risk [66]. Cardioprotection is thought to be secondary to antithrombotic effects as well as inhibition of atherosclerosis from LDL cholesterol [67]. In a 2025 study by Sui et al., moderate to heavy alcohol intake, defined as greater than 112 g per week, was associated with greater CIMT [68]. Additionally, heavy alcohol intake has been associated with increased risk of PAD [69]. Mechanistically, alcohol can promote atherosclerosis through oxidative stress, hypertension, dyslipidemia, and direct cardiotoxicity.

### 5.4. Electronic Cigarette Use

While tobacco cigarette use is a traditionally known cardiovascular risk factor, significantly less is known about the use of electronic cigarettes. The nicotine in electronic cigarettes is thought to lead to atherosclerotic plaque development. Through sympathetic nerve activity, nicotine is also thought to result in a pro-inflammatory environment. The flavoring component of electronic cigarettes is thought to lead to endothelial cell dysfunction as well as reactive oxygen species formation. Flavorants such as acetoin can lead to the release of interleukins, resulting in an inflammatory state. Electronic cigarettes also contain fine heavy metal particles thought to lead to vascular inflammation and endothelial dysfunction [70]. A 2023 cross-sectional study used an in vitro model to assess the proatherogenic changes found in blood monocytes in patients who used tobacco cigarettes, patients who used electronic cigarettes, and patients who were non-smokers. They determined that the proatherogenic changes were highest in the tobacco group, significantly less in electronic cigarette users, and the least in individuals who abstained from both [71].

### 5.5. Endocrine and Reproductive Vulnerability

Women with a history of adverse pregnancy outcomes such as gestational hypertension, preeclampsia, preterm delivery, or gestational diabetes are at higher risk for CAD, even if they are thought to traditionally be at low risk of ASCVD. A history of preeclampsia or small-for-gestational-age infants is thought to result in endothelial dysfunction, thereby triggering atherosclerosis [72]. In addition, women with polycystic ovary syndrome are at increased risk of subclinical atherosclerosis, likely due to elevated levels of circulating androgens along with increased underlying inflammation [73].

### 5.6. Chronic Kidney Disease (CKD)

With worsening CKD, risk of CAD progressively increases [74]. Notably, stable angina is a classical initial presentation for CAD. However, in patients with CKD, particularly with a glomerular filtration rate less than <45 mL/min/1.73 m^2^, acute myocardial infarction is more likely to be their initial presentation rather than simply stable angina [75]. With regard to screening for CAD, exercise testing and pharmacologic stress testing are less accurate in patients with CKD [76]. The mechanisms by which CKD stimulates atherosclerosis are still being investigated. A possible mechanism could be CKD-mineral bone disorder, which results in vascular calcifications. In patients with CKD, there is also thought to be modification of LDL and HDL, such as glycation and oxidation, which are associated with pathogenic pathway activation, leading to inflammation. In addition, CKD is marked by systemic inflammation, and patients frequently have elevated CRP and cytokine levels. There are also increased circulating levels of CD14+16+ monocytes that have been proposed to result in vascular damage. Traditionally, nephrotic-range proteinuria was thought to create a proatherogenic lipid profile. However, recent research has shown that even milder degree of proteinuria/microalbuminuria has been associated with ASCVD [77].

### 5.7. Chronic Obstructive Pulmonary Disease (COPD)

COPD leads to airflow limitations and pulmonary inflammation. Many patients with COPD have comorbid cardiovascular disease. Importantly, the majority of COPD in industrialized countries can be traced back to tobacco use, which is a risk factor for the development of concurrent ASCVD [78]. It is hypothesized that COPD causes systemic inflammation and subsequent endothelial dysfunction, which makes these patients prone to increased cardiovascular risk [79]. In addition, there is increased platelet activation and atherothrombosis [78]. COPD patients may also have higher CAC scoring in comparison to individuals without COPD [80]. Inhaled corticosteroid use has been associated with decreased cardiovascular events, likely due to a decrease in systemic inflammation as well as inhibition of immune cell recruitment and adhesion to the arterial wall [78].

### 5.8. Rheumatologic Conditions

Systemic inflammation seen in rheumatologic conditions such as rheumatoid arthritis (RA) and systemic lupus erythematosus (SLE) is thought to also contribute to cardiovascular risk [81]. For instance, arterial stiffening is seen in both RA and SLE. In SLE, IL-6 and CRP are thought to be inflammatory mediators implicated in arterial stiffening [82]. The use of biologic agents such as tumor necrosis factor alpha inhibitors is thought to decrease the rate of aortic stiffness in RA [83]. Cardiovascular risk assessment is therefore paramount in patients with rheumatologic conditions, and comorbid cardiovascular conditions should be closely monitored [84].

## 6. Environmental and Infectious Risk Factors

### 6.1. Air Pollution

Outdoor air pollution can be either natural or anthropogenic in origin [85]. Chronic exposure to fine particulate matter (PM2.5) has been associated with increased vascular inflammation, endothelial dysfunction, and arterial remodeling [86]. In particular, particulate matter exposure is thought to increase the levels of circulating sphingolipids that promote the production of lipoproteins and also atherosclerotic plaque. Particulate matter also promotes the production of reactive oxygen species and reactive nitrogen species. The increase in reactive oxygen species is thought to decrease the amount of circulating nitric oxide, inhibiting vascular relaxation. Increased oxidative stress results in increased oxidation of LDL, which accumulates in the intima. Exposure to certain airborne particles can lead to the production of TNF-alpha and IL-6, which promote chemotaxis and vascular wall damage. Air pollution also leads to increased expression of cellular adhesion molecules, which are critical in plaque formation. Particulate matter exposure can lead to increased levels of CD4 and CD8 T-cells, leading to atherosclerotic disease progression [85]. A meta-analysis by Akintoye et al. confirmed a significant correlation between PM2.5 exposure and CIMT, indicating a direct environmental contribution to subclinical atherosclerosis [87]. Similar findings were concluded in a study by Liu et al., in which particulate matter exposure was associated with increased CIMT as well [88]. Another type of air pollution, black carbon, can be correlated with traffic-related combustion products, specifically diesel particles. Wilker et al. concluded an association between black carbon exposure and CIMT in elderly men [89].

### 6.2. Noise Pollution

Transportation noise has been getting recent attention as well and has been associated with atherosclerosis. The release of stress hormones in response to noise has been postulated to play a role in the development of ASCVD [90]. Stress hormones, in turn, lead to the release of inflammatory markers such as IL-6 and IL-1β [91]. Noise pollution is also thought to affect DNA methylation and epigenetic patterns, upregulating inflammatory pathways [92].

### 6.3. Heavy Metal Exposure

Exposure to heavy metals such as arsenic and mercury has increased due to advances in industrialization and an increase in anthropogenic activities. This exposure results in oxidative stress, reactive oxygen species formation, and an inflammatory cascade that results in atherosclerosis. Public health interventions aimed towards curbing exposure are essential [93].

### 6.4. Helicobacter Pylori Infection

Helicobacter pylori, particularly strains expressing cytotoxin-associated gene A (CagA), has been implicated in promoting vascular inflammation. Diomedi et al. found that H. pylori infection was associated with increased CIMT [94]. Rahmani et al. further reported that H. pylori infection doubled the odds of myocardial infarction, reinforcing the pathogen’s relevance in cardiovascular risk stratification [95]. In a study involving patients with a first-time ischemic stroke, chronic H. pylori infection was associated with higher risk of stroke caused by small-artery occlusion [96]. In a large meta-analysis consisting of over 6000 H. pylori-positive patients and over 7000 H. pylori-negative patients, individuals positive for H. pylori had increased CIMT [97].

### 6.5. Respiratory Pathogens

Chronic infections with Chlamydia pneumoniae and Mycoplasma pneumoniae have also been linked to atherosclerosis. Jalili et al. conducted a meta-analysis demonstrating significant associations between these pathogens and subclinical as well as clinical vascular disease, likely due to sustained low-grade inflammation and endothelial injury [98].

### 6.6. Human Immunodeficiency Virus (HIV) Infection

With modern anti-retroviral therapy, HIV is now a chronic condition. It is hypothesized that HIV proteins may affect immune and vascular cells, triggering atherogenesis. In addition, immunodeficiency could result in translocation of the gut microbiome, resulting in plaque formation. Even latent HIV infection is associated with inflammatory markers such as CRP and IL-6, which could trigger atherogenesis [99].

## 7. Physical and Oral Indicators of Atherosclerosis

Emerging evidence suggests that certain physical and oral signs may serve as accessible, non-invasive markers of underlying atherosclerosis. Among these, diagonal earlobe crease (DELC), also known as Frank’s sign, and chronic periodontitis have gained increasing attention as clinical indicators of subclinical and overt cardiovascular disease.

### 7.1. Frank’s Sign

Frank’s sign, a diagonal ear lobe crease extending from the tragus to the earlobe’s edge, was first reported in 1973 and has since been studied as a potential physical marker of vascular disease. While its prevalence increases with age, several studies—including a systematic review by Pacei et al. have demonstrated an independent association between the presence of Frank’s sign and CAD, cerebrovascular disease, and PAD [100]. Its simplicity and visibility make it a potentially useful clinical cue for further cardiovascular evaluation, especially in patients without overt risk factors.

### 7.2. Periodontitis

Periodontal disease is a chronic inflammatory condition of the gingival and supporting tissues that has consistently been associated with systemic inflammation and atherosclerosis. Periodontal disease shares many risk factors with atherosclerotic disease progression, including increased inflammatory biomarkers such as CRP, increased levels of circulating leukocytes, vascular adhesion molecules, and pro-inflammatory cytokines [101]. Interestingly, microbiota from the periodontal area have been found in atherosclerotic plaque, raising the idea of possible bacterial translocation [102]. In addition, periodontal pathogens are able to oxidize lipoproteins, leading to atherogenesis [103]. Certain periodontal bacteria, such as *P. gingivalis*, can even infect vascular endothelial cells, leading to mitochondrial fragmentation and creation of reactive oxygen species [104]. Periodontitis is also thought to result in vascular calcification [105]. Lastly, *P. gingivalis* is also thought to result in plaque destabilization through the production of matrix metallopeptidase 9 [101]. A recent meta-analysis by Lu et al. found that periodontitis significantly elevated ASCVD risk, particularly in patients with metabolic syndrome [106]. *Porphyromonas gingivalis* is the dominant bacterial flora in subgingival plaque biofilms and is thought to trigger an inflammatory cascade and plaque formation in the vasculature [107]. A meta-analysis by Wang et al. also demonstrated increased PAD risk in individuals with periodontal disease [108].

## 8. Vulnerable Populations

Certain populations of individuals have higher rates of ASCVD in the absence of traditional risk factors. One group includes South Asians, defined as individuals from Sri Lanka, Pakistan, India, Bangladesh, and Nepal. It has been demonstrated that South Asians experience premature ASCVD. For instance, South Asians have increased rates of acute myocardial infarction at younger ages [109]. A study by Nanda demonstrated that Indian Asians aged 40 years and under experiencing an acute myocardial infarction were mostly of normal body weight, had a relatively low incidence of comorbidities, such as hypertension and diabetes, and largely did not participate in excessive cigarette smoking. They were more likely to have certain physical exam findings as well such as corneal arcus [110]. In addition, as demonstrated by Datey and Nanda, in Asian Indians, latent diabetes mellitus can be unmasked after acute myocardial infarction [111].

The Mediators of Atherosclerosis in South Asians Living in America (MASALA) study recruited South Asians to understand the relationship between non-traditional cardiovascular risk factors and atherosclerosis [112]. While traditionally high-density lipoprotein cholesterol (HDL-C) was thought to attenuate CAC density, in South Asians, there was a positive association between HDL-C concentration and CAC density. In addition, higher waist circumference was associated with lower CAC density in both men and women [113]. Furthermore, previous work by the Kulkarni and Nanda group has demonstrated that Asian Indians have highly atherogenic small, dense low-density lipoprotein levels, which predispose them to CAD [114]. In addition, as detailed above, Lp(a) levels are traditionally associated with atherosclerotic disease. In a 2019 study using MASALA study data, Lp(a) levels were higher in South Asian men compared to Whites, Hispanics, and Chinese-Americans. The INTERHEART Lp(a) study demonstrated elevated Lp(a) levels and increased risk of myocardial infarction in South Asians, Southeast Asians, Latin Americans, Chinese, and Europeans. The odds ratio for myocardial infarction was highest in South Asians, as demonstrated in a study by Pare et al. [115]. In addition, while Lp(a) levels are traditionally correlated with aortic valve calcifications, this association was not seen in South Asians in a 2020 study [116].

A 2019 study using data from the MASALA study analyzed CAC incidence and progression. They found that CAC progression was higher in South Asian men compared to South Asian women. In addition, they found that South Asian men had greater changes in CAC from initial to subsequent measurements compared to black, Latino, and Chinese men [117].

Psychosocial factors have also been implicated in the development of atherosclerosis in South Asians. Particularly, in a MASALA study, anxiety and depression were associated with increased CIMT in men, while stress was associated with increased CIMT in women [118].

A recent study by Gupta et al. examined North Indian patients presenting to the hospital for their first ST-elevation myocardial infarction. They noted approximately 25% of patients lacked traditional risk factors such as hypertension, hyperlipidemia, tobacco use, or diabetes mellitus, which was higher than what is seen in Western cohorts. This study underscored the notion that a lack of risk factors does not necessarily confer low risk of atherosclerotic disease. On the contrary, these patients likely have undetected vascular inflammation or genetic predisposition [119].

In a study by Markovitz et al., Asian Indians were noted to have elevated fibrinogen and platelet activation levels compared to white individuals. It is thought that hemostatic factors such as fibrinogen increase risk of thrombosis, thereby serving as a possible explanation for the increased coronary risk observed in Asian Indians [120]. In another study by Markovitz, South Indians with diabetes and CAD had higher levels of platelet activation as well [121].

**Figure 1:**
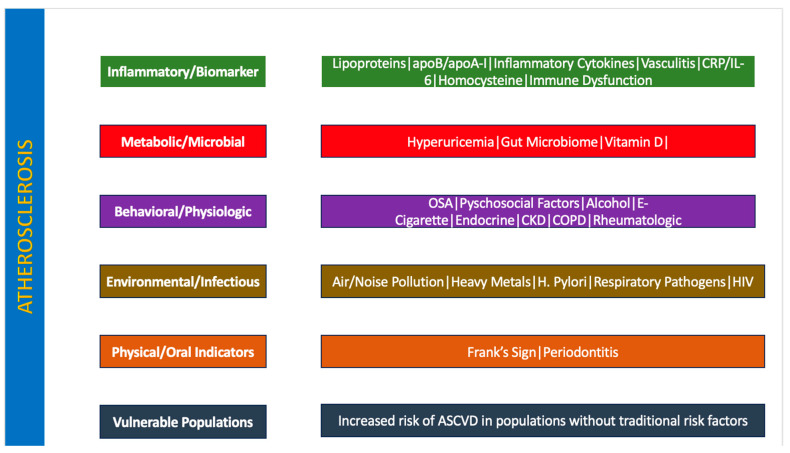
Summary of Non-Traditional Risk Factors for Atherosclerosis: A summary of non-traditional risk factors of atherosclerosis grouped by the following categories: inflammatory/biomarker (green), metabolic/microbial (red), behavioral/physiologic (purple), environmental/infectious (brown), physical/oral indicators (orange), vulnerable populations (gray). This framework underscores measurable and potentially modifiable contributors beyond traditional lipid-centered risk models.

**Figure 2:**
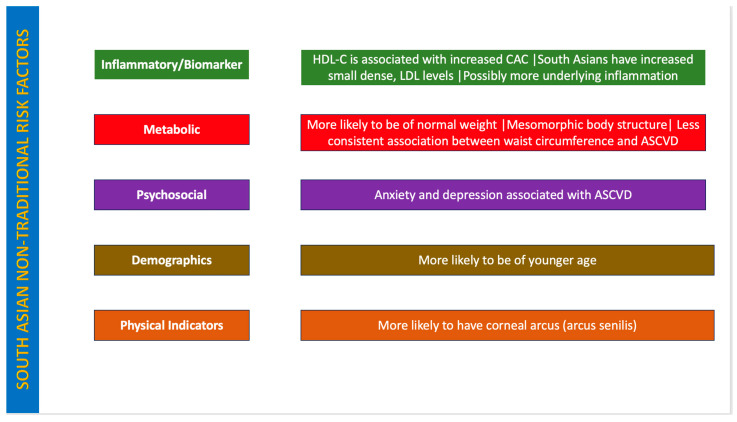
Summary of non-traditional risk factors for atherosclerosis in South Asians: A summary of non-traditional risk factors for atherosclerosis in South Asians. High density lipoprotein-cholesterol (HDL-C), Coronary artery calcium (CAC), Atherosclerotic cardiovascular disease (ASCVD).

Table 1 and Table 2 show high-yield non-traditional risk factors for atherosclerosis and laboratory testing to stratify atherosclerotic risk, respectively. Figure 1 and Figure 2 depict a summary of non-traditional risk factors for atherosclerosis and a summary of these factors in South Asians, respectively.

## 9. Clinical Implications & Integration

### 9.1. Incorporating Non-Traditional Risk Factors into Practice

Several non-traditional risk factors—such as Lp(a), OSA, and periodontal disease—are clinically detectable using accessible tools, including laboratory assays, sleep studies, and oral health evaluations. Visual signs like Frank’s sign offer additional low-cost, non-invasive opportunities to prompt deeper risk evaluation.

### 9.2. Actionable vs. Emerging Factors

Some risk factors, such as OSA, depression, and H. pylori infection, are modifiable with existing therapies or lifestyle changes. Others, including microbiome profiles, cytokine polymorphisms, and apolipoprotein genotypes, remain primarily research-based but hold promise for precision risk stratification in the future.

### 9.3. Relevance to Preventive Care and Counseling

Primary care and cardiology providers can enhance risk communication by educating patients about these lesser-known contributors during routine visits. Intermediate-risk patients may benefit most from expanded evaluations that go beyond traditional models. Depression screening and oral health assessment, for example, are simple additions that may improve early detection and outcomes.

### 9.4. Toward Future Risk Assessment Tools

Next-generation risk stratification tools may incorporate molecular and behavioral data, including biomarkers, polygenic risk scores, and microbiome sequencing. These integrative approaches could lead to highly personalized cardiovascular risk profiles that better reflect the disease’s multifaceted origins.

## 10. Future Directions & Research Gaps

### 10.1. Standardization and Measurement

A significant barrier to implementation is the lack of standardized definitions and diagnostic criteria for many non-traditional risk factors, including depression, periodontitis, and gut dysbiosis. Harmonization across studies will be essential to improve reproducibility and comparability. Developing consensus frameworks could facilitate translation of emerging biomarkers, such as apolipoprotein B and inflammatory cytokine profiles, into clinical guidelines.

### 10.2. Prospective Multi-Ethnic Cohorts

Current literature often suffers from geographic and demographic limitations. Future research should prioritize large, longitudinal, multi-ethnic cohorts to confirm associations between metabolic, microbial, and psychosocial risk factors across diverse populations. In addition, continued research on vulnerable populations will offer more insight into non-traditional cardiovascular risk factors in these individuals.

### 10.3. Therapeutic Target Development

Emerging therapeutic targets include Lp(a), IL-1 and IL-6, and microbiota-influenced metabolic cascades. Randomized controlled trials will be necessary to determine whether modifying these factors translates into reduced atherosclerotic progression or event rates.

### 10.4. Integrative Models and Machine Learning

Artificial intelligence and systems biology approaches may revolutionize cardiovascular risk prediction by integrating genomic, proteomic, microbiomic, and environmental data. Machine learning models trained on multiomic datasets could yield precise, individualized risk algorithms.

## 11. Conclusions

Atherosclerosis is a multifactorial disease extending far beyond traditional lipid-centric frameworks. This review consolidates evidence linking inflammatory, infectious, metabolic, psychosocial, and physical markers—such as OSA, H. pylori, hyperuricemia, and periodontal disease—to increased ASCVD risk. Many of these risk factors are modifiable through established interventions or behavioral modification, offering tangible opportunities for prevention. Traditional scoring systems such as the Framingham and ASCVD calculators often underestimate risk in certain populations; integrating non-traditional factors like lipoprotein(a), psychosocial stress, and oral health measures could bridge that gap. In addition, special attention to vulnerable populations is crucial in understanding non-traditional risk factors. Future cardiovascular care will likely evolve toward multi-dimensional, precision-guided risk prediction grounded in molecular, behavioral, and environmental data, moving from population-based models toward truly individualized prevention and treatment.

## Figures and Tables

**Table 1:** High-Yield Non-Traditional Risk Factors for Atherosclerosis.

Domain	Risk Factor	Mechanism	Clinical Relevance
**Inflammation & Biomarker-Based**	ApoB/ApoB:ApoA-I ratio	Stronger predictor of ASCVD than LDL-C;	Routinely measurable; may guide lipid-lowering therapy decisions
	Cytokine polymorphisms (IL-1, TNF-α variants)	Variability in inflammatory response, mixed causal links	Primarily research; potential for future personalized medicine
	Vasculitides	Accelerated atherosclerosis due to inflammation	Screening/management should be integrated into CVD care
	CRP and IL-6	Accelerated atherosclerosis due to inflammation	Screening/management should be integrated into CVD care
	Homocysteine	Accelerated atherosclerosis due to inflammation	Screening/management should be integrated into CVD care
**Metabolic & Microbial**	Hyperuricemia	Independent predictor of coronary calcification and CAD	Serum uric acid is easily testable; target for intervention debated
	Gut microbiota dysbiosis/TMAO	Alters lipid metabolism and vascular tone	Emerging research may guide personalized interventions
	Vitamin D deficiency	Associated with increased CIMT and plaque burden	Screening feasible; supplementation efficacy mixed
**Behavioral & Physiologic**	Obstructive sleep apnea	Increases sympathetic drive, oxidative stress, and vascular dysfunction	Diagnosable via sleep studies; treatable with CPAP
	Depression, anxiety, psychosocial stress	Linked with higher mortality and impaired cardiovascular outcomes	Screening/management should be integrated into CVD care
	Alcohol use	Dose-dependent cardiotoxicity and oxidative stress	Counseling and behavioral modification
	E-Cigarette Use	Proatherogenic changes	Counseling and behavioral modification
	Chronic kidney disease	Linked with increased atherosclerotic disease	Screening/management should be integrated into CVD care
	Chronic obstructive pulmonary disease	Linked with increased atherosclerotic disease	Screening/management should be integrated into CVD care
	Rheumatologic conditions	Linked with increased atherosclerotic disease	Screening/management should be integrated into CVD care
**Environmental & Infectious**	Air pollution (PM2.5)	Associated with endothelial dysfunction and CIMT progression	Public health and advocacy implications
	Noise pollution	Release of stress hormones in response to noise	Public health and advocacy implications
	Heavy metal exposure	Associated with inflammation and atherosclerosis	Public health implications
	Helicobacter pylori infection	Associated with CIMT, impaired FMD, and MI risk	Treatable infection; potential risk modifier
	Chlamydia pneumoniae & Mycoplasma pneumoniae	Chronic infection driving low-grade inflammation	Research-emerging; possible risk modifier
	HIV infection	Chronic inflammation/immunodeficiency, predisposing to atherosclerosis	Screening/management should be integrated into CVD care
**Physical & Oral Indicators**	Frank’s sign (earlobe crease)	Associated with CAD, PAD, and stroke	Low-cost, visible bedside cue
	Periodontitis	Microbial translocation & inflammation	Dental hygiene and periodontal care as prevention adjunct

ApoA-I = Apolipoprotein AI; ApoB = Apolipoprotein B; ASCVD = Atherosclerotic Cardiovascular Disease; CAD = Coronary Artery Disease; CIMT = Carotid Intima-Media Thickness; CPAP = Continuous Positive Airway Pressure; CRP = C-Reactive Protein; CVD = Cardiovascular Disease; FMD = Flow-Mediated Dilation; HIV = Human Immunodeficiency Virus; IL-1 = Interleukin-1; IL-6 = Interleukin-6; LDL-C = Low-Density Lipoprotein Cholesterol; MI = Myocardial Infarction; PAD = Peripheral Artery Disease; PM2.5 = Particulate Matter 2.5; TMAO = Trimethylamine-N-oxide; TNF-α = Tumor Necrosis Factor-Alpha.

**Table 2:** Laboratory Testing to Stratify Atherosclerotic Risk.

Domain	Lab Test	Interpretation	Therapy
**Inflammation & Biomarker-Based**	High Sensitivity CRP	Greater than 3 mg/L indicates high inflammatory risk	Address the underlying inflammatory process
	Homocysteine	Normal < 15 µmol.	Folic acid and vitamin B12
	Uric Acid	Normal range between 3.5 and 7.2 mg/dL	If symptomatic, urate-lowering therapy (xanthine oxidase inhibitor, uricosuric agents, etc.)
**Metabolic & Microbial**	Vitamin D	Normal 40–80 ng/mL	Vitamin D supplementation
	Fibrinogen	Normal range between 200 and 400 mg/dL.	Address the underlying inflammatory process
	Glomerular filtration rate	Glomerular filtration rates consistent with advanced CKD have increased atherosclerotic risk.	If no other contraindications, renin–angiotensin–aldosterone system blockers or SGLT2 inhibitors can offer nephroprotection [122].
	Urine creatinine	>30 mg/g of urinary creatinine is thought to have increased atherosclerotic risk	Address the underlying kidney pathology.
**Nontraditional Lipids**	ApoB	Greater than 100 mg/dL indicates higher atherosclerotic risk	Standard dyslipidemia treatment (e.g., statins, ezetimibe, proprotein convertase subtilisin/kexin type 9 inhibitors) and aggressively modifying lifestyle risk factors.
	Lp(a)	>50 mg/dL is thought to represent the risk-enhancing cutoff	Standard dyslipidemia treatment as above. Emerging targeted treatments in the pipeline.

ApoB = Apolipoprotein B; CKD = Chronic Kidney Disease; CRP = C-Reactive Protein; Lp(a) = Lipoprotein (a); SGLT2 = Sodium-Glucose Transporter 2.

## Data Availability

No new data were created or analyzed in this study. Data sharing is not applicable to this article.
